# Laser-Assisted Lipolysis: A Promising Alternative to Traditional Liposuction

**DOI:** 10.1007/s00266-025-05056-8

**Published:** 2025-08-06

**Authors:** Alexander Manzano-Finol, Heliana Parra-Pelosi, Gabriel Cubillos-Valencia, Juan Urdaneta, Diego Rivera-Porras, Maricarmen Chacín, Valmore Bermúdez

**Affiliations:** 1 Endocrine and Metabolic Diseases Research Center, School of Medicine, Universidad del Zulia, Maracaibo, 4002 Venezuela; 2 Clínica de Obesidad y Envejecimiento, Bogotá, Colombia; 3 Mental Health Clinic, Insight Mental Wellness, San Antonio, Texas 78217 USA; 4 Departamento de Productividad e Innovacion, Universidad de la Costa, 080001 Barranquilla, Atlantico, Colombia; 5 Facultad de Ciencias de la Salud, Universidad Simón Bolívar, 080001 Barranquilla, Colombia; 6 Centro de Investigaciones en Ciencias de la Vida, Universidad Simón Bolívar, 080001 Barranquilla, Colombia; 7 Internal Medicine Department, University of New Mexico, Albuquerque, USA

**Keywords:** Laser-assisted lipolysis, Traditional liposuction, Fat removal, Multi-wavelength system, Minimally invasive laser treatments

## Abstract

**Background:**

Laser-Assisted Lipolysis (LAL) has emerged as a modern advancement in body contouring, offering solutions to limitations of traditional liposuction such as prolonged recovery, increased bruising, and limited skin-tightening effects. By using targeted laser energy to liquefy fat before removal, LAL minimizes tissue trauma, enhances skin retraction, and improves overall aesthetic outcomes.

**Objective:**

To provide a comprehensive review of the historical evolution, current technological advances, comparative methodologies, limitations, and future directions of LAL in cosmetic surgery.

**Methods:**

A narrative literature review was conducted, incorporating peer-reviewed articles, FDA-cleared devices, and clinical studies. Topics included the historical progression of LAL since its inception in the late 1990s, comparative analyses between LAL and traditional techniques, evaluation of laser wavelengths (e.g., 1064, 1320, 1444, and 1470 nm), and examination of innovations such as dual-wavelength systems, robotic-assisted delivery, AI-guided planning, and radiofrequency-assisted methods.

**Results:**

LAL shows clinical advantages including faster recovery times, reduced postoperative pain, and improved skin tightening, especially in small to moderate fat reduction areas. Dual- and multi-wavelength lasers, pulse modulation, and fiber-optic temperature sensing improve precision and safety. Comparative studies show LAL achieves better outcomes than traditional liposuction in certain contexts, particularly for skin appearance and submental fat reduction. However, variability in laser parameters, higher procedural costs, and limitations in autologous fat reuse remain concerns. Research also highlights promising applications of LAL in large-volume fat removal and potential bariatric contexts.

**Conclusion:**

LAL has evolved into a valuable technique in modern aesthetic surgery. While not a replacement for traditional liposuction in all cases, it offers meaningful benefits as a stand-alone or adjunct procedure. Continued innovations, such as nanotechnology, biodegradable fibers, and AI-assisted planning, may further enhance its effectiveness, precision, and personalization in future body contouring practices.

*Level of Evidence V* This journal requires that authors assign a level of evidence to each article. For a full description of these Evidence-Based Medicine ratings, please refer to the Table of Contents or the online Instructions to Authors www.springer.com/00266

## Introduction

Liposuction has long been a cornerstone of body contouring procedures in cosmetic surgery. Traditional liposuction techniques, while effective, often come with significant drawbacks such as prolonged recovery times, extensive bruising, and potential complications. In recent years, LAL has emerged as a promising alternative that addresses many of these concerns, revolutionizing the approach to fat removal and body sculpting [1].

LAL utilizes targeted laser energy to liquefy adipose tissue prior to removal, offering several advantages over traditional liposuction methods. This innovative technique aims to minimize trauma to surrounding tissues, reduce postoperative discomfort, and enhance skin-tightening effects. These benefits result not only in shorter recovery times and a decreased risk of complications but also enhance aesthetic outcomes. The procedure typically involves the insertion of a small laser fiber through tiny incisions, which delivers energy directly to the adipocytes, causing them to rupture and liquefy for easier removal [2]. By selectively targeting adipose tissue while preserving surrounding structures, LAL represents a significant advancement in the field of body contouring [3].

The integration of laser technology into liposuction procedures has opened new possibilities for achieving more precise and less invasive fat removal. This review will shed light on how LAL is changing the landscape of cosmetic surgery. As we progress through this comprehensive analysis, we will uncover the scientific principles underlying this technique, its clinical applications, and the ongoing research that continues to refine its efficacy [3].

## Historical Background

The journey of LAL along with other techniques from concept to clinical application is a testament to the advancements in medical technology and the continuous pursuit of improved patient outcomes in cosmetic surgery [4]. As we delve into the historical background of this innovative technique, it is essential to understand the context in which it emerged and the key milestones that shaped its development.

Laser technology was first introduced in the field of cosmetic surgery in the 1980s, primarily focusing on skin resurfacing and hair removal. The concept of utilizing laser energy for fat removal began to take shape in the late 1990s, marking the inception of LAL as a novel approach to body contouring [4, 5]. This innovative idea represented a significant departure from traditional liposuction techniques, which relied solely on mechanical disruption and suction of adipose tissue [5].

The pioneering work in this field can be attributed to Dr. Rodrigo Neira and colleagues, who in 1999 first reported the use of low-level laser therapy to emulsify adipose tissue [6]. Their groundbreaking study demonstrated that exposure to 635 nm diode laser energy could create temporary pores in adipocyte membranes, leading to the release of stored lipids. This discovery laid the foundation for the development of LAL as we know it today [6].

Dr. Neira’s research opened a new avenue for fat removal techniques, prompting further investigations into the potential of laser energy in body contouring procedures. The initial findings suggested that laser treatment could facilitate fat removal with less trauma to surrounding tissues, potentially reducing postoperative pain and recovery time. This prospect was particularly appealing in an era where minimally invasive procedures were gaining popularity among both patients and practitioners [7].

Following Dr. Neira’s work, several key milestones marked the progression of LAL technology:

1. In 2006, a significant breakthrough occurred with the FDA approval of the first laser device specifically designed for LAL. Cynosure’s SmartLipo system, which utilized a 1064 nm Nd laser, marked the official entry of laser technology into the realm of clinical liposuction procedures. This approval paved the way for wider adoption of the technique in cosmetic practices across the USA [8, 9].
2. The year 2007 saw the introduction of the 1320 nm wavelength laser for lipolysis. This development showcased improved efficacy in fat liquefaction and collagen stimulation. The 1320 nm wavelength demonstrated a higher affinity for fat and water, allowing for more efficient energy absorption and potentially enhanced skin-tightening effects. This advancement highlighted the potential of LAL not only for fat removal but also for overall skin quality improvement [8].
3. In 2009, a significant leap forward was achieved with the development of multi-wavelength systems combining 1064 nm and 1320 nm lasers. This innovation aimed to optimize both fat removal and skin-tightening effects simultaneously. The dual-wavelength approach allowed practitioners to tailor treatments more precisely, addressing both deep and superficial fat layers while promoting collagen remodeling in the dermis. This development marked a shift toward more comprehensive body contouring solutions [10].
4. The year 2012 brought about the introduction of 1470 nm diode lasers for LAL. These lasers exhibited higher absorption by water and fat, thereby improving both efficacy and safety. The enhanced absorption profile of the 1470 nm wavelength allowed for more efficient energy delivery to target tissues, potentially reducing the risk of thermal injury to surrounding structures. This advancement represented a significant step toward optimizing the balance between effective fat removal and patient safety [11].
5. In 2015, the FDA clearance of 1060 nm diode lasers for noninvasive fat reduction expanded the application of laser technology in body contouring. This milestone broadened the spectrum of laser-assisted body contouring techniques, offering patients a completely noninvasive option for fat reduction. The introduction of noninvasive laser treatments highlighted the versatility of laser technology in addressing various patient needs and preferences in body sculpting [12].
6. These milestones reflect the rapid advancement of LAL technology, with each new development focusing on enhancing efficacy, safety, and patient outcomes. The transition from single-wavelength systems to multi-wavelength approaches and the exploration of different laser types exemplify the continuous endeavor to optimize this technique [12].

As we reflect on the historical journey of LAL, it is clear that this technique has come a long way from its initial conception. The continuous refinement of laser technologies and treatment protocols has transformed LAL from an experimental procedure to a well-established technique in cosmetic surgery. The ongoing research and development in this field promise further advancements, potentially revolutionizing the approach to body contouring and fat removal in the years to come [13].

## Current Trends and Advances

The field of LAL continues to evolve rapidly, with ongoing research and technological advancements pushing the boundaries of what is possible in body contouring.

Liposuction techniques have advanced significantly over the last generation of devices. Current liposuction technologies include suction-assisted lipectomy and ultrasound-, power-, laser-, water-, and radiofrequency-assisted methods. Recent studies in LAL have emphasized optimizing treatment parameters, combining different wavelengths, and exploring new applications [14]. These research efforts aim to enhance the efficacy, safety, and versatility of LAL procedures. However, some noteworthy techniques in the field include:

Dual-wavelength systems: Recent research indicates that integrating 1064 nm and 1470 nm wavelengths may yield superior fat liquefaction and skin tightening compared to single-wavelength treatments. The combination of these wavelengths allows for simultaneous targeting of different tissue components, potentially leading to more comprehensive and efficient body contouring results. A study by DiBernardo et al. demonstrated that dual-wavelength systems could achieve up to 17% more skin contraction compared to single-wavelength treatments, highlighting the potential benefits of this approach [15].

Low-level multifrequency laser lipolysis: This approach utilizes lasers at wavelengths of 533, 650, and 980 nanometers, each providing specific benefits: vasoconstriction, adipocyte lysis, and skin tightening, respectively. The combined effects result in minimized blood loss, significant fat extraction with minimal tissue trauma, and neo-collagen formation. A recent study by Cubillos-Valencia et al. (2024) also showed this approach as effective and safe after surgery procedure to treat breast ptosis demonstrating a correction rate of 90% for breast ptosis after the third month [16].

Pulse modulation: Studies have shown that pulse-modulated laser delivery can provide better control over thermal effects and improve overall safety compared to continuous wave lasers. Pulsed laser delivery allows for precise energy deposition while minimizing heat accumulation in surrounding tissues. A recent investigation by Kantola et al. (2019) found that pulse-modulated laser treatments resulted in a 30% reduction in postoperative edema compared to continuous wave laser treatments, suggesting improved patient comfort and faster recovery times [17].

Photoacoustic lipolysis: This emerging technique merges laser energy with ultrasound to enhance fat cell disruption and augment overall efficacy. The combination of these two energy modalities creates a synergistic effect, potentially leading to more efficient fat removal and improved skin tightening. Preliminary studies have shown promising results, with photoacoustic lipolysis demonstrating up to 25% greater fat reduction compared to traditional LAL alone [18].

Robotic-assisted laser lipolysis: This innovative approach integrates robotic systems with laser lipolysis devices to enhance the precision and consistency of treatments. Robotic assistance can help standardize the movement of the laser fiber, ensuring uniform energy delivery across the treatment area. Early clinical trials have shown that robotic-assisted laser lipolysis can achieve up to 15% more consistent fat reduction compared to manual techniques, potentially leading to improved aesthetic outcomes [19, 20].

Fiber-optic temperature sensing: Real-time temperature monitoring during laser lipolysis allows for optimized energy delivery while enhancing safety. By integrating fiber-optic temperature sensors into laser cannulas, practitioners can precisely control the thermal effects of the treatment, minimizing the risk of overheating and potential complications. A study by Chia et al. (2018) demonstrated that fiber-optic temperature sensing could reduce the incidence of thermal injuries by up to 40% compared to conventional laser lipolysis techniques [21].

Artificial intelligence-guided treatment planning: Employing AI algorithms to analyze patient anatomy can facilitate the optimization of treatment parameters, yielding individualized results. AI-guided systems can process pretreatment imaging data to create personalized treatment maps, suggesting optimal laser settings and treatment patterns based on the patient’s unique anatomy and desired outcomes [22]. Preliminary research indicates that AI-guided treatments can lead to up to 20% improvement in patient satisfaction scores compared to standard treatment protocols [23].

Radiofrequency-based technologies: Driven by demand for nonexcisional alternatives to address lax skin following liposuction, various energy-based technologies have become available. Among these, helium plasma radiofrequency (RF; Renuvion, Apyx Medical, Clearwater, Fla.) and bipolar RF (BodyTite, InMode, Irvine, Calif.) have gained prominence [24]. The helium plasma RF device precisely controls the delivery of heat to the tissue with minimal thermal spread. This application provides rapid heating to 85 °C with near-instantaneous tissue cooling; at 85 °C, causing the collagen to contracts 60% in 0.044 seconds without requiring external temperature monitoring of the skin [25, 26]. The bipolar RF device provides subdermal tissue coagulation by bulk heating to 65 °C–70 °C by RF electrodes having direct contact with the tissue; at 65 °C, collagen contracts by 40% in approximately 2.5 minutes, whereas at 75 °C, collagen contracts 40% in approximately 0.42 s. Due to the method of action of bulk heating, external temperature monitoring of the skin is required to manage the surface temperature of the skin to prevent adverse events (AEs) such as burns [27].

Power-assisted liposuction: Power-assisted liposuction utilizes a mechanized cannula that vibrates rapidly, which helps to break up fat cells more efficiently, making the suction process easier and potentially reducing surgeon fatigue. This method does not involve the use of heat or lasers, and it primarily focuses on the mechanical disruption and removal of fat tissue. Clinical studies comparing the PAL system to manual liposuction found that PAL aspirates 31% more volume per minute, leading to a 35% reduction in procedure time. Precise PAL is proven to reduce surgeon fatigue by 49%, offering a safer and more precise procedure. These benefits result in superior outcomes and faster healing and recovery rates for patients [28, 29].

### Comparison of Approaches

A comparative analysis of various methodologies highlights their distinct characteristics and potential advantages:

1. Laser-Assisted vs. Traditional Tumescent Liposuction: LAL generally results in less bruising, a faster recovery, and potential skin-tightening effects compared to traditional methods [4]. Ali et al. reported that LAL patients experienced 40% less postoperative pain and returned to normal activities 3 days earlier on average compared to traditional liposuction patients [30]. However, traditional tumescent liposuction still holds advantages in terms of larger-volume removal and has a more established long-term safety profile [31, 32]. Specifically, in small-volume areas such as submental fat accumulation, LAL demonstrated significantly greater fat reduction compared to traditional liposuction (*p* = 0.001). At the 2-week follow-up visit, submental fat thickness in the lipolysis group showed a significantly greater reduction than in the liposuction group (*p* = 0.05). Additionally, 39% of patients in the liposuction group reported bruising lasting more than one week, whereas no significant adverse effects were reported in the lipolysis group, aside from minimal pain and discomfort following the procedure. Improvement in skin appearance was also significantly greater in the lipolysis group (*p* = 0.001) [33].
2. 1064 nm Nd vs. 1444 nm Diode Lasers: The 1064 nm wavelength demonstrates better penetration depth, making it suitable for larger-volume fat removal, particularly in areas with thicker subcutaneous fat layers. Conversely, the 1444 nm wavelength shows higher absorption by fat and water, potentially enhancing efficiency for smaller areas and improving skin tightening. A comparative study found that the 1,064-nm wavelength requires three times more energy than the 1,444 nm wavelength to remove the same amount of fat tissue [34].
3. Single vs. Multi-wavelength Systems: Single-wavelength systems are simpler to operate and may be more cost-effective, making them an attractive option for practitioners new to LAL. However, multi-wavelength systems offer greater versatility, effectively targeting both fat removal and skin tightening simultaneously balancing efficacy and control, which might be advantageous in cases where both deep and superficial improvements are needed. A study by Kim et al. [35] concluded that multi-wavelength systems achieved an average of 18% greater overall patient satisfaction compared to single-wavelength treatments, primarily due to improved skin quality outcomes [36].
4. Low-level multifrequency Diode Lasers vs 1064 nm Diode Laser: This multifrequency laser approach offers comprehensive benefits, including reduced complications, enhanced recovery, and improved aesthetic outcomes with a faster postsurgical recovery and improved patient comfort compared with conventional procedures. Cubillos et al (2024) described it as a large adipose volume extraction procedure, offering low complication rates like 1064 nm wavelength laser-assisted lipolysis. These significant adipose volume extractions are categorized as mega-volume and gigantic-volume liposuction procedures. Concluding the potential consideration for low-level multifrequency laser approach as a bariatric intervention with improved aesthetic sequelae [37, 38].
5. Radiofrequency-based technologies: With the introduction of energy during liposuction with LAL or ultrasound-assisted liposuction (UAL), many patients are left with undesirable skin laxity [39–42]. While both LAL and UFAL are effective for fat reduction and skin tightening, radiofrequency-assisted liposuction (RFAL) has demonstrated significant improvements in skin laxity and contouring, with high patient satisfaction and minimal complications [43–47]. Studies have shown that RFAL can effectively treat conditions like breast ptosis and cellulite by improving skin tightness and reducing laxity [43, 44]. LAL, while effective for fat reduction, may not achieve the same degree of skin tightening as RFAL, particularly in areas with significant skin laxity.

The current trends and advances in LAL reflect a dynamic and rapidly evolving field. As research continues to uncover the optimal parameters and techniques for various clinical scenarios, practitioners can look forward to increasingly sophisticated and effective tools for body contouring. The integration of advanced technologies, such as robotics and AI, promises to further refine and personalize treatments, potentially leading to superior outcomes and higher patient satisfaction [48].

Additionally, it is crucial for practitioners to stay informed about these developments and critically evaluate the emerging evidence to provide the most effective and safer treatments for their patients. The future of LAL looks bright, with ongoing innovations poised to further solidify its position as a leading technique in modern body contouring (Tables 1 and 2).

**Table 1:** Comparison of approaches in LAL

Approach	Description	Advantages	Limitations
Traditional liposuction [32]	Mechanical removal of fat using suction	Effective for large volume fat removal Well-established technique	Longer recovery time Less precision in contouring Limited skin-tightening effect
Single-wavelength laser lipolysis [32]	Use of a single laser wavelength for fat melting	Improved skin tightening Reduced recovery time compared to traditional liposuction	Limited versatility May not be optimal for all tissue types
Multi-wavelength laser lipolysis [49]	Use of multiple laser wavelengths	Enhanced versatility Better targeting of different tissue depths Improved skin tightening	More complex equipment Requires more expertise to optimize settings
Noninvasive laser fat reduction [50, 51]	Transcutaneous laser application without incisions	No downtime Reduced risk of complications Suitable for patients averse to surgery	Limited efficacy compared to invasive methods May require multiple treatments Not suitable for large volume fat removal
Combined energy-based approaches [15, 18]	Integration of laser with RF or ultrasound	Synergistic effects on fat reduction and skin tightening Versatile treatment options	Increased complexity of procedures Potential for higher costs
Low-level multifrequency laser [37]	Use of multiple low-laser wavelengths	Synergistic effects on blood vessel sealing fat reduction and skin tightening Suitable for large volume fat removal	More complex equipment Requires more expertise to optimize settings

**Table 2:** Comparison of liposuction technique and volume of fat removal

Study	Liposuction technique	Sample size	Volume described in study	Primary outcome measure	Key findings
Cubillos G, et al. [37]	Laser-assisted lipolysis (LAL)	101	Large-volume liposuction: 5000 L aspirate Mega-volume liposuction: 8 000-L aspirate Giganto-volume liposuction: 12 L or more	Weight-related changes before and after the first three postoperative months Surgical complications within 30 days	Significant decreases in BMI, %TWL, and %EBMIL during follow-up at months 1, 2, and 3
R. Albin et al. [52]	Tumescent liposuction Tumescent liposuction + ultrasonic liposuction	181	Large-volume liposuction: 5000-L aspirate	Case series of large volume liposuction description	No correlation between the aspirate volume and the calculated blood loss One case of deep venous thrombosis Two cases of pulmonary emboli No deaths
G W Commons et al. [53]	Traditional liposuction techniques Ultrasound-assisted liposuction Superwet technique	631	Total aspirate volumes ranged from 3 to 17 liters (94,5% under 10 liters)	Total aspiration volumes	80% of patients maintained stable weights one year after surgery. No serious complications were experienced
G Giugliano et al. [54]	Single large volume liposuction Superwet technique	30	Mean aspirate volume: 3540 mL (range 2550–4670) Net lipid loss of 2.7 ± 0.7 kg (mean ± SD)	Insulin sensitivity Inflammatory markers	Decreased insulin sensitivity Reduced concentrations of proinflammatory markers Increased serum levels of adiponectin and HDL-cholesterol
Youssef Saleh et al.[55]	Standard liposuction Tumescent infiltration	60	Average aspirate amount of 6000 mL	Total aspirate volumes Rate of complications	Safety in large-volume liposuction with desirable morphological and hematological changes
Hanna Habib et al. [56]	Tumescent liposuction	15	The range: 5–12 liters Mean volume: 7.36 ± SD of 1.84L	Fasting insulin changes after three months following large volume liposuction	Significant improvement in fasting insulin levels with aspiration of large amounts of subcutaneous abdominal fat
S. Y. Giese et al. [57]	Ultrasound-assisted lipoplasty Standard liposuction technique Superwet technique	14	Mean fat aspirate: 6733 mL (range 4675 to 8825 mL), Total estimated surgical fat removal: 6.1 ± 1.2 kg (range 3.8 to 7.3 kg)	Fasting insulin levels, weight, systolic and diastolic blood pressure, heart rate, and body circumferences	Improvements in body weight, systolic blood pressure, and fasting insulin levels observed four months after the procedure had been maintained at 10 to 21 months after lipoplasty

*SD* Standard deviation, *BMI* Body mass index, *%TWL* percentage total weight loss, *%EBMIL* Percentage excess BMI loss

## Limitations, Controversies and Future

### Limitations

*Thermal damage to the skin and surrounding tissues* The absorption of laser energy by water in the skin can lead to thermal injury, which may manifest as skin blistering or burns [3, 58, 59]. This risk necessitates careful control of laser parameters and may limit the effectiveness of the procedure in terms of fat reduction depth and overall lipolysis efficiency [59, 60]. However, Chia et al, presented 1,000 consecutive cases of laser-assisted liposuction and suction-assisted lipectomy managed with local anesthesia, where no major complications or mortalities were observed. There were three burns, two infections, one hematoma, and one seroma. A total of 73 secondary procedures were performed (7.3%) [21].

*Lack of standardization in laser energy settings* The variability in outcomes depending on the laser wavelength and system used has been widely shown. Different wavelengths, such as 980 nm, 1064 nm, and 1320 nm, have been approved by the FDA, but there is no consensus on which is superior for specific clinical applications [3]. This variability can lead to inconsistent results and complicates the comparison of LAL with conventional liposuction techniques [2]^.^

*Unclear evidence in terms of efficacy over tumescent liposuction* The literature also highlights the need for more comparative studies to better understand the relative benefits and limitations of LAL compared to other liposuction techniques [2]. Where superiority in efficacy is shown in some scenarios [34], others question its practicality. Additionally, since LAL effectively emulsify fat to ease extraction; however, they disrupt the fat cells to a level that might not be suitable for autologous lipoinjection [60–65].

*Procedure times and additional costs* As with any new technology, there is a significant learning curve associated with LAL [65], although the slope is relatively steep in experienced hands [66]. Sun Y, et al. noted a long operative time when laser lipolysis was employed in the abdomen even in the hands of the most-skilled surgeons [67]. As earlier generation and most contemporary LAL devices require two steps, first for the tissue to be treated with the laser followed by a separate aspiration step [65, 66]. The innovation of dual-functioning cannulas, allowing simultaneous laser firing and suction, resolves this issue. Finally, the cost of additional laser equipment is a barrier to entry for some practitioners [68].

### Controversial Topics

#### Noninvasive vs. Minimally Invasive Laser Treatments

A debate persists about the effectiveness of noninvasive laser treatments versus minimally invasive LAL. While noninvasive treatments eliminate downtime, concerns arise regarding their efficacy in substantial fat reduction. A study by Lee et al. compared and combined minimally invasive laser-assisted with noninvasive lipolysis laser system, obtaining, on average 20% and 6% fat reduction effects respectively. In addition, the combination of two systems resulted in a fat reduction of about 35 % and the results of blood tests and biopsies showed no abnormalities in safety. Interestingly, patient satisfaction rates were similar across both groups, implying that treatment choice may hinge on individual preferences and treatment goals [51].

#### Future Directions

- Research Gaps

There is a critical need for long-term studies comparing LAL with traditional liposuction. These studies should investigate sustained fat reduction, skin quality enhancement, and long-term patient satisfaction. Such research will provide a comprehensive understanding of the durability of results and assist clinicians in offering more informed recommendations. Furthermore, long-term research could uncover late-onset complications or benefits not apparent in shorter-term assessments [32].

- Optimization of Energy Delivery

Refining laser parameters, such as pulse modulation and wavelength combinations, is essential to improve efficacy while minimizing side effects. Studies focusing on optimizing laser settings to achieve fat melting and skin tightening while preventing thermal damage to surrounding tissues are crucial. Enhanced control of laser energy can lead to more consistent results across varying patient demographics [69].

- Tissue-specific Targeting

Innovations in selective laser systems that can precisely target adipose tissue without damaging adjacent structures are critical. By enhancing laser precision, these advancements could reduce recovery times and postoperative complications. New technologies may include chromophores or photosensitizers that accumulate exclusively in fat cells to minimize collateral damage and enhance targeting [70].

- Nanotechnology-Enhanced Laser Lipolysis

The integration of nanoparticles into LAL could significantly improve energy absorption and specificity, resulting in more efficient fat melting with lower energy usage. Nanoparticles could also offer real-time feedback during procedures, enabling more personalized and precise treatment plans [71].

- Biodegradable Laser Fibers

Developing absorbable laser fibers could reduce postoperative complications, like infection, by eliminating the need for fiber removal. These fibers could also be designed to release therapeutic agents during degradation, enhancing healing or delivering localized pain relief [72].

- Personalized Treatment Planning

The utilization of AI and 3D imaging could revolutionize treatment planning by analyzing patient-specific factors such as anatomy, fat distribution, and skin elasticity. AI-driven systems could predict outcomes and optimize treatment parameters, leading to higher patient satisfaction and improved consistency [48].

## Conclusion

LAL has emerged not only as a compelling alternative to traditional liposuction but also as a complementary tool to traditional liposuction, offering numerous potential benefits that are reshaping the field of body contouring. This innovative technique has demonstrated the ability to reduce recovery time significantly, allowing patients to return to their daily activities more quickly compared to conventional liposuction. The procedure’s capacity for improved skin tightening addresses one of the primary concerns associated with traditional fat removal methods, potentially leading to more aesthetically pleasing results, especially in areas prone to skin laxity. Furthermore, the enhanced precision offered by LAL enables practitioners to target specific areas with greater accuracy, potentially resulting in more sculpted and refined outcomes. At the same time as it offers advantages in fat emulsification, these are limited by risks of thermal injury, lack of standardized laser settings, and unclear efficacy over conventional liposuction. While studies report low major complication rates and burns, inconsistent outcomes persist due to variable wavelengths and energy parameters.

The evolution of laser technologies in this field has been remarkable, progressing from single-wavelength systems to more sophisticated multi-wavelength platforms. This technological advancement has significantly improved both the efficacy and versatility of LAL. Multi-wavelength systems allow practitioners to tailor treatments more effectively to individual patient needs, addressing various tissue depths and targeting both fat reduction and skin tightening simultaneously. Moreover, innovative technologies such as nanotechnology-enhanced procedures or biodegradable fibers could also further refine the approach to body contouring. This versatility has expanded the range of treatable areas and improved the overall quality of results.

Current research in the field of LAL is primarily focused on three key areas. First, there is a concerted effort to optimize treatment parameters, including energy levels, pulse durations, and wavelength combinations. This optimization aims to maximize fat reduction and skin-tightening effects while minimizing the risk of complications. Second, researchers are exploring innovative laser types that could offer improved efficacy or safety. Finally, there is a growing interest in integrating advanced technologies, such as artificial intelligence and 3D imaging, to enhance treatment planning and execution. As these research and innovation continue to evolve, LAL is poised to redefine the standards of aesthetic medicine, offering safer, more effective, and personalized treatment options for patients. Although LAL has been used successfully as a sole procedure for body contouring, some physicians assert that LAL is not a substitute for conventional liposuction, but a complement to it.
